# Variations in Proline Content, Polyamine Profiles, and Antioxidant Capacities among Different Provenances of European Beech (*Fagus sylvatica* L.)

**DOI:** 10.3390/antiox13020227

**Published:** 2024-02-12

**Authors:** Marko Kebert, Srđan Stojnić, Milena Rašeta, Saša Kostić, Vanja Vuksanović, Mladen Ivanković, Miran Lanšćak, Anđelina Gavranović Markić

**Affiliations:** 1 Institute of Lowland Forestry and Environment, University of Novi Sad, 21000 Novi Sad, Serbia; srdjan.stojnic@uns.ac.rs (S.S.); sasa.kostic@uns.ac.rs (S.K.); 2 Department of Chemistry, Biochemistry, and Environmental Protection, Faculty of Sciences, University of Novi Sad, 21000 Novi Sad, Serbia; milena.raseta@dh.uns.ac.rs; 3 Faculty of Agriculture, University of Novi Sad, 21000 Novi Sad, Serbia; vanja.vuksanovic@polj.uns.ac.rs; 4 Division for Genetics, Forest Tree Breeding and Seed Science, Croatian Forest Research Institute, 10450 Jastrebarsko, Croatia; mladeni@sumins.hr (M.I.); miranl@sumins.hr (M.L.); 5 Division for Silviculture, Croatian Forest Research Institute, 10450 Jastrebarsko, Croatia; andelina@sumins.hr

**Keywords:** beech, provenance, polyamines, spermidine, proline, antioxidant capacities

## Abstract

International provenance trials are a hot topic in forestry, and in light of climate change, the search for more resilient beech provenances and their assisted migration is one of the challenges of climate-smart forestry. The main aim of the study was to determine intraspecific variability in European beech (*Fagus sylvatica* L.) among 11 beech provenances according to total antioxidant capacities estimated by various assays, such as DPPH (2,2-diphenyl-1-picrylhydrazyl), ABTS (2,2′-azino-bis-(3-ethylbenzothiazoline-6-sulfonic) acid), FRAP (ferric reducing antioxidant power) assay, and radical scavenging capacity against nitric oxide (RSC-NO assays), as well as osmolyte content, primarily individual polyamines (putrescine, spermidine, and spermine), and free proline content. Polyamine amounts were quantified by using HPLC coupled with fluorescent detection after dansylation pretreatment. The highest values for radical scavenger capacity assays (ABTS, DPPH, and FRAP) were measured in the German provenances DE47 and DE49. Also, the highest NO inhibition capacity was found in the provenance DE49, while the highest content of proline (PRO), total phenolic content (TPC), and total flavonoid content (TFC) was recorded in DE47. The Austrian AT56 and German provenance DE49 were most abundant in total polyamines. This research underlines the importance of the application of common antioxidant assays as well as osmolyte quantification as a criterion for the selection of climate-ready beech provenances for sustainable forest management.

## 1. Introduction

Ongoing global climate change, characterized by increased mean temperature, extreme summer heat, and altered seasonal precipitation patterns, as well as declined precipitation during the prolonged summer season, is one of the main causes of soil aridity and drought in forest ecosystems [1,2,3]. Although beech presents the most prominent and widely distributed deciduous tree species across Europe, high evolutionary pressure imposed by climate change might detrimentally affect the future distribution range of this species [4,5]. Indeed, the vulnerability of beech forests to limited water availability and their potential migrations have been described in many studies [6,7,8]. For example, recent species distribution models indicated a change in the current beech stand location with the prediction that the European beech will reduce its elevational distribution and exhibit a significant upward shift towards higher, more suitable elevation during the second half of the 21st century in the territory of Serbia, whereas European beech stands below 500 m are threatened and might disappear [9].

Due to the high economic and ecological importance of beech, the biological response of this species to upcoming climate change is one of the key issues of sustainable forest management. A potential approach in climate-smart forestry involves evaluating beech provenances’ adaptability to extreme climatic events, such as heat and drought, followed by human-assisted movement—so-called “assisted migration”—in order to accelerate translocation of trees’ genetic material into more climatically suitable areas, as well as to complement local seed sources through gene flow and alternation in the genetic composition [10,11]. Numerous studies have established and confirmed high variability in physiological and morpho-anatomical traits among various populations of beech species, emphasizing the fact that populations originating from warmer climates are less vulnerable to abiotic stress than those growing under more favorable conditions [12,13]. Indeed, a recent study clearly evidenced that beech provenances originating from warmer sites in Southern Europe were more resistant to drought-induced embolism than Northern European provenances originating from more mesic sites [14]. However, research with a focus on biochemical traits and the evaluation of oxidative stress variability in beech provenances has been seldom reported. Previous research has addressed the significance of various metabolites and oxidative stress parameters in tracking the acclimation of beech trees of different origin to environmental stress conditions by using a non-targeted metabolomic approach [13,15,16,17,18,19], while this study is primarily focused on the estimation of beech inter-specific variation with respect to main polyamines by employing a targeted metabolic approach.

Essentially, oxidative stress arises when the capacity of the antioxidant defense machinery to scavenge reactive oxygen species (ROS) is exceeded by uncontrolled excessive generation of ROS, either during plant respiration or as a result of impairment of the light and dark phases of photosynthesis under limited amounts of an electron-accepting reductant agent such as NADPH, which consequently drastically disturbs redox homeostasis [20]. This phenomenon is more prominent in times of abiotic and biotic stress and can have detrimental effects, including the oxidation of important plant cell bio-polymers and causing their malfunction [20]. To prevent oxidative damage, plants have developed complex systems of enzymatic and nonenzymatic antioxidants that can modulate the redox balance within plant cells and scavenge ROS [21].

Due to its antioxidant potential and chaperone and osmoprotective activities, proline is undoubtedly one of the most studied osmolytes related to abiotic stress in plants [22,23]. Increased resistance to various abiotic stressors, including heat [24], drought [25], or a combination of heat and drought [26], or drought and rising CO_2_ [27], as well as the presence of heavy metals [28,29] or xenobiotics [30] in various woody plant species, has been linked to increased endogenous proline levels. As a multifunctional amino acid, proline not only helps plants adapt to a variety of biotic and abiotic stressors, but it also functions as a signaling molecule that controls gene expression and plant development, modifies redox balance, and modulates defense against pathogens [23]. The research hypothesizes that specific beech provenances exhibiting higher proline levels may be more resilient to climate extremes, including heat waves and drought.

Another noteworthy class of osmolytes that significantly contributes to the amelioration of abiotic and biotic stress factors in woody plants is polyamines (PAs). PAs are ubiquitous polycations that stabilize negatively charged molecules like lipid membranes and nucleic acids due to their positive charge at physiological pH; therefore, they modulate ion channels, which makes them crucial for salinity and thermotolerance [31,32,33,34]. In addition to their well-known antioxidant qualities, polyamines as ubiquitous aliphatic polycationic compounds may also act pro-oxidatively [34,35]. Namely, during their catabolism and by the action of polyamine oxidases, they generate hydrogen peroxide, which can, in a Fenton-like reaction, further produce hydroxyl radicals as initiators of lipid peroxidation; on the other hand, hydrogen peroxide as a signaling molecule may cause stomata closure and prevent water loss during drought stress. Intriguingly, polyamines stimulate the biosynthesis of nitric oxide [36], which, as a signal molecule, is also capable of initiating stomata closure but is also a key molecule in thermotolerance and initiation of a hypersensitive reaction during biotic stress in plants [37]. Recently, high inter- and intraspecific variability of individual polyamines was reported in various woody plant species in urban areas [38]. The novelty of this research lies in the fact that this is the first study to examine provenance-dependent variability of individual polyamine levels in European beech species.

Therefore, the primary goal of the research was to assess the variability of different beech provenances by monitoring the concentrations of major osmolytes such as proline and polyamines (putrescine, spermidine, and spermine), total phenolics, and flavonoids, as well as various total antioxidant leaf capacities determined by various biochemical assays (FRAP, ABTS, and DPPH assay). Additionally, this study aimed to determine the intra-specific heterogeneity of PA profiles in beech seedlings and to establish a relationship among osmolytes, such as proline and polyamines, and the different antioxidant capacities of leaf extracts of different beech provenances. Finally, the authors aimed to identify the “climate-ready” beech provenance candidates that may be employed in future reforestation programs through assisted migration in order to address the challenges imposed by climate changes and adapt forests to them.

## 2. Materials and Methods

### 2.1. Provenance Experiment Setup

**Figure 1:**
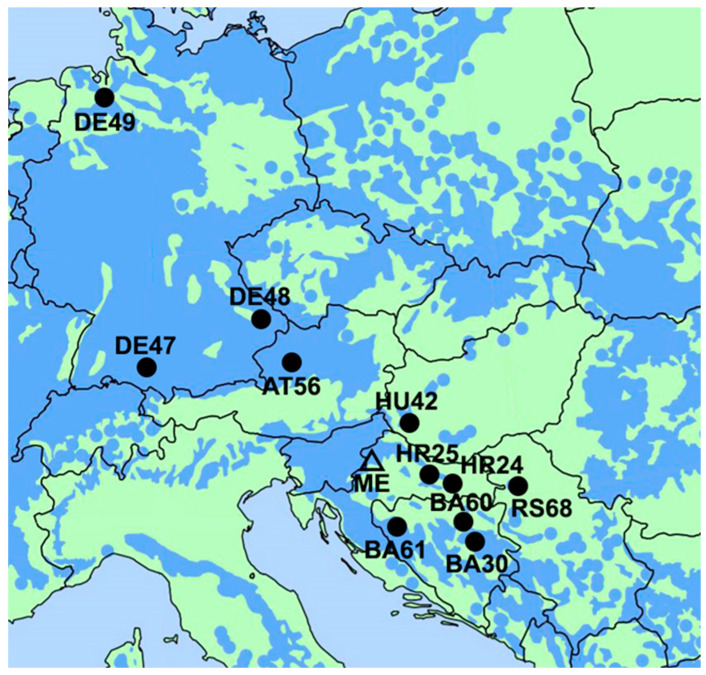
Natural distributional range of European beech (blue area) [45] and origins of inspected provenances marked with a full circle. The triangle designates the location of the experiment. Details of abbreviated provenances are listed in Table S1.

The European beech provenance trial located at the Medvednica Mountain in Croatia (latitude 45°52′, longitude 15°55′; elevation 450 m above sea level) was initiated in 2007. It was established within the framework of the COST Action E52–Evaluation of the Genetic Resources of Beech for Sustainable Forestry, contributing to the pan-European network of provenance trials. More information about provenance trial experimental design and climatic conditions was given by Stojnić et al. [39]. The study involved 11 provenances (AT56, BA30, BA60, BA61, DE47, DE48, DE49, HR24, HR25, HU42, RS68) that originated from northern Germany and through Central Europe (Austria and Hungary) to the southern parts of the beech distribution range in the Balkan region (Bosnia and Herzegovina, Croatia, and Serbia) (Figure 1; Table S1). The climatic information about provenances’ origin sites was obtained from the ClimateChart application for the period 1980–2015 [40].

Ellenberg’s climate quotient (EQ) is given in Table S1. Ellenberg’s climate quotient (EQ) was calculated according to the following equation: EQ = 1000 × (T_July_/P_ann_), where T_July_—mean air temperature in July (°C) and P_ann_—annual sum of precipitations (mm) [4]. It is considered that EQ values below 20 represent the most favorable habitats for beech growth. The vitality of the species begins to decline as EQ increases between 20 and 30, while beech forests disappear in the areas where EQ > 30 [4,41,42]. Soil characteristics of beech forest on Medvednica Mountain have previously been reported [43], wherein the soil type was determined to be cambisol; the specific soil characteristics for this experiment are provided in supplementary material S2 [44]. Leaf material for the analyses was collected in mid-August 2021 from three healthy and dominant trees from each provenance. Fully sun-exposed, fully expanded, and hardened leaves situated in the upper third part of the crown were sampled from the same orientation using telescope scissors. After harvesting, the leaf samples of selected beech provenances were rapidly frozen in liquid nitrogen and transported to the laboratory. In the laboratory, leaf samples were freeze-dried at −70 °C in a lyophilizer (model alpha 1-4LSC basic, Martin Christ, Osterode am Harz, Germany) and powdered in liquid nitrogen prior to the analysis.

### 2.2. Measurements of Osmolyte Accumulation

#### 2.2.1. Quantification of Free Proline (PRO)

Following ninhydrin reagent treatment, free PRO was evaluated spectrophotometrically using a rapid colorimetric method, with slight changes, as outlined by Bates et al. [46]. After mixing 1 mL of sulfosalicylic acid (3% *w*/*v*) with about 20 mg of freeze-dried leaf material, the mixture was centrifuged for 10 min at 4000 rpm.

The supernatant (0.7 mL) was mixed with a solution of acid ninhydrin (2.5% ninhydrin in a mixture of glacial acetic acid, distilled water, and 85% orthophosphoric acid in a 6:3:1 ratio). The mixture was boiled at 95 °C for an hour and then cooled in an ice bath. After vigorous vortexing to extract the resulting pinkish PRO-ninhydrin complex, 2 mL of toluene was added, and the absorbance at 520 nm was measured. 

Using a standard calibration curve, the concentration of free PRO was calculated and represented as µmol/gram dry weight (µmol g^−1^ DW).

#### 2.2.2. HPLC Quantification of Polyamines (PAs)

Approximately 30 mg of lyophilized beech leaf material (dry weight–DW) underwent extraction using a solution of 4% perchloric acid (*v*/*v*) in a volume ten times that of the sample. The resulting homogenate was cooled on ice for 1 h and then centrifuged at 15,000× *g* for 30 min. Following this, supernatant samples, along with standard solutions containing putrescine (PUT), spermidine (SPD), and spermine (SPM), were subjected to dansylation treatment following the procedure outlined by Scaramagli et al. [47] and Biondi et al. [48]. The resulting dansylated derivatives were extracted using toluene, then dried and reconstituted in acetonitrile. The separation and quantification of polyamines (PAs) were conducted using high-performance liquid chromatography (HPLC) coupled with fluorescent detection (Nexera XR, Shimadzu, Kyoto, Japan), employing a reverse-phase C18 column (Spherisorb ODS, 2.5-μm particle diameter, 4.6 × 250 mm, Waters, Wexford, Ireland) and a programmed acetonitrile–water gradient [47,48]. The results of PA quantification were expressed as nmol per gram of dry weight (nmol g^−1^ DW).

### 2.3. Evaluation of Antioxidant Potential and Quantification of Total Phenolic and Flavonoid Contents

Beech leaf extracts were derived from freeze-dried leaf material to evaluate antioxidant activity. In 2 mL test tubes, ethanolic extracts were formulated by mixing approximately 0.2 g of freeze-dried leaf powder with 2 mL of 80% ethanol. The resulting supernatants, obtained through a 20 min centrifugation at 13,200 rpm at 4 °C, were utilized for 2,2-diphenyl-1-picrylhydrazyl (DPPH), 2,2′-azino-bis-(3-ethylbenzothiazoline-6-sulfonic) acid (ABTS), radical scavenging capacity-nitric oxide (RSC-NO), and ferric reducing antioxidant power (FRAP) assays, in addition to the quantification of total phenolic and flavonoid content.

#### 2.3.1. DPPH and ABTS Assays

The ability of the extracts to neutralize the 2,2-diphenyl-1-picrylhydrazyl (DPPH) radical was assessed using the protocol described by Arnao [49]. In this procedure, 10 µL of the sample was mixed with 100 µL of 90 µM DPPH solution in MeOH and 190 µL of MeOH. The absorbance was measured after a 5 min incubation period at 515 nm.

For the ABTS assay, the conversion of the blue-green cation radical ABTS^•+^ to its colorless form was monitored spectrophotometrically. The method followed the procedure outlined by Miller and Rice-Evans [50]. Subsequently, 10 µL of leaf extract was added to 290 µL of the prepared ABTS solution, and the absorbance of the sample was measured at 734 nm following a 5 min incubation at room temperature.

#### 2.3.2. NO Assay

The inhibition of the nitric oxide radical (NO^•^) was assessed using the Griess diazotization process, following the protocol outlined by Hensley et al. [51]. The reaction mixture consisted of 15 µL of the extract, 250 µL of 10 mmol/L sodium nitroprusside (SNP, Na_2_[Fe(CN)_5_NO]), and 250 µL of phosphate buffer, pH 7.4. After an incubation period of 90 min at room temperature under constant light, 500 µL of Griess reagent, comprising a 0.2% solution of N-(1-naphthyl)-ethylenediamine dihydrochloride and a 2% solution of sulfanilamide in 4% phosphoric acid, was added. The extent of inhibition was determined by measuring the absorbance of the resulting chromophore at 546 nm.

#### 2.3.3. FRAP Assay

The FRAP (ferric reducing antioxidant power) assay followed the protocol described by Benzie and Strain [52]. The FRAP reagent, freshly prepared, consisted of a solution containing 10 mmol/L TPTZ (2,4,6-tri(2-pyridyl)-s-triazine) in 40 mmol/L HCl, 0.02 mmol/L FeCl_3_x6H_2_O, and acetate buffer (pH 3.6) in a ratio of 10:1:1. In this assay, 10 µL of each extract was mixed with 225 µL of the FRAP reagent and 22.5 µL of distilled water. The absorbance was measured after 6 min at 593 nm, with Trolox used to establish the standard curve.

#### 2.3.4. The Total Phenolic Content (TPC)

The total phenolic content (TPC) was determined using the Folin–Ciocalteu (FC) method, as described by Singleton et al. [53]. This method involves the spectrophotometric detection of phenolic compounds forming a colored complex with the FC reagent. For each extract or standard solution, 25 µL was combined with 125 µL of 0.1 mol/L FC reagent, and after 10 min, 100 µL of 7.5% sodium carbonate was added and thoroughly mixed. The absorbance was measured at 760 nm, and TPC was expressed in milligrams of gallic acid equivalents (GAE) per gram of dry weight (mg GAE g^−1^ DW).

#### 2.3.5. The Total Flavonoid Content (TFC)

The total flavonoid content (TFC) of the extracts was assessed via a colorimetric method based on the formation of a flavonoid–aluminum complex, following the protocol outlined by Chang et al. [54]. For this assay, 30 μL of the extract or standard solution was mixed with 90 μL of methanol, along with 6 μL of 10% AlCl_3_, 6 μL of 1 mol/L CH_3_COONa, and 170 μL of distilled water. The absorbance was then measured at 415 nm, and the TFC was determined in milligrams of quercetin equivalents (QE) per gram of dry weight (mg QE g^−1^ DW).

The radical scavenging capacity (RSC) against DPPH, ABTS, and NO radicals, as well as the reducing power using the FRAP assay, were evaluated by constructing a standard curve of Trolox. The results were quantified and expressed as millimoles (mmol) of Trolox equivalents per gram of dry weight of plant material (g DW), denoted as mmol TEAC g^−1^ DW.

### 2.4. Statistical Analysis

Quantified data were presented with mean and standard deviation, while variations among provenances were tested with one-way ANOVA and Pearson’s correlations, which were visually presented on a correlation matrix. Likewise, Principal Component Analysis (PCA) and dendrogram hierarchical clustering were used to group provenance following tracked biochemical parameters. The ANOVA results were interpreted using the Fisher (F) test and their statistical significance levels (*p*).

All statistical data processing was performed with an R environment. Basic descriptive statistics, as well as ANOVA, were calculated via the “rstatix” R package, while the “den-extend” R package was used for clustering analyses. Finally, the “ggplot2” R package [55,56,57] was used for other visual representations.

## 3. Results

**Figure 2:**
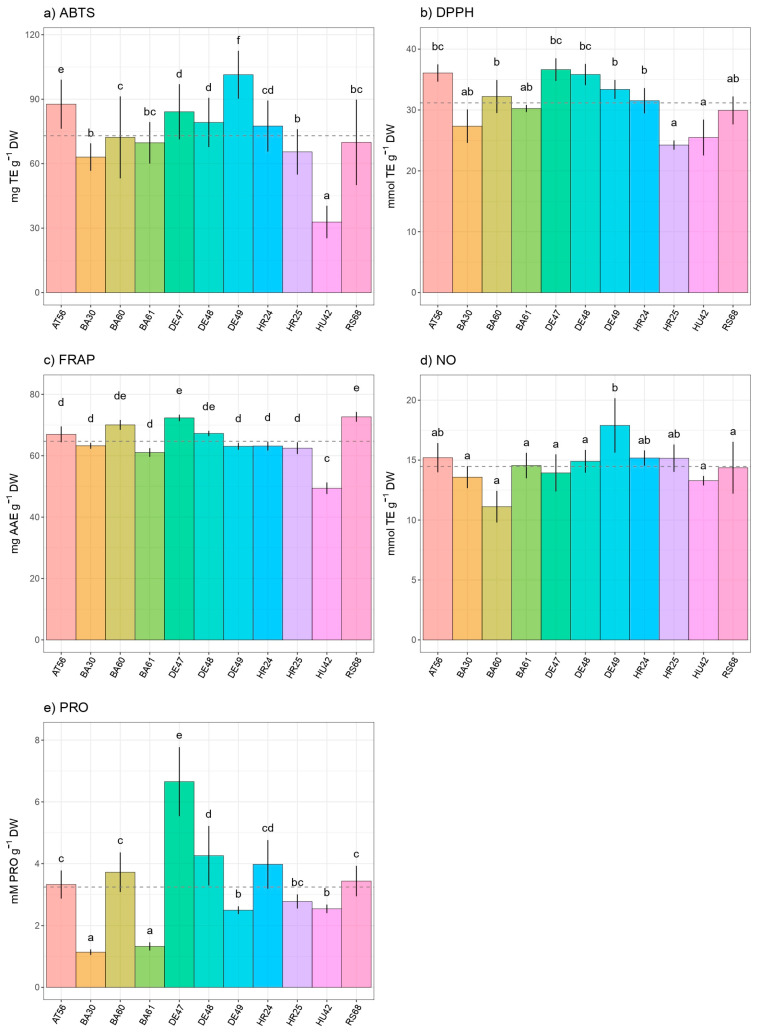
Intraspecific variability in European beech (*Fagus sylvatica* L.) leaves regarding: (**a**) radical scavenger capacity against ABTS radical (2,2′-azinobis-3-ethylbenzothiazoline-6-sulfonic acid); (**b**) radical scavenger capacity against DPPH radical; (**c**) ferric reducing antioxidant power (FRAP) assay; (**d**) neutralization of nitric oxide radical; (**e**) free proline content (PRO). Distinct lowercase letters denote significant differences observed among different species, as determined by Tukey’s honestly significant difference (HSD) post hoc test (*p* ≤ 0.05). The data are presented as the mean ± standard deviation (SD).

High interprovenance variation of *Fagus sylvatica* regarding total antioxidant capacity estimated by the ABTS assay was recorded (Figure 2a). The lowest Trolox equivalent (TE g^−1^ DW) value was measured in Hungarian provenance HU42 (32.8 mg TE g^−1^ DW), while the highest Trolox equivalent value was found in German provenance DE49 (101.4 mg TE g^−1^ DW).

The values of the DPPH test ranged from 24.2 mg TE g^−1^ DW, found in Croatian provenance HR25, to 36.6 mg TE g^−1^ DW, recorded in German provenance DE47 (Figure 2b). Variability in provenances within the same country was not observed for the DPPH test, except in the case of beech leaves from Croatia. In this instance, the measured scavenger capacity against DPPH in the Croatian provenance HR24 was significantly higher compared to the HR25 provenance. European beech provenances DE47 and DE48 stood out with the strongest total antioxidant capacity based on radical scavenger capacity against DPPH, while provenances HU42 and HR45 showed the lowest total antioxidant properties.

The highest reducing capacity estimated by the FRAP test was measured in the leaves of Serbian provenance RS68 (72.7 mg AAE g^−1^ DW), while the lowest values were found in the leaves of Hungarian provenance HU42 (49.4 mg AAE g^−1^ DW) (Figure 2c). Low inter- and intraprovenance variability with respect to reducing capacity has been observed among selected European beeches.

On the other hand, the ability of extracts to neutralize NO radicals varied depending on the provenance (Figure 2d). The lowest scavenging capacity against NO radicals was detected in the leaves of beech trees originating from Bosnia in BA60 (11.1 mg TE g^−1^ DW), while the highest NO inhibition capacity was found in the leaves of provenance DE49 (17.9 mg TE g^−1^ DW). The obtained NO-inhibiting capacity did not significantly differ amongst different provenances within the same country.

Significant inter- and intraprovenance (within the same country) variabilities in proline content were observed. German provenance DE47 had the highest proline content (6.65 mM PRO g^−1^ DW) in beech leaves, while Bosnian provenance BA30 exhibited the lowest proline amounts (1.13 mM PRO g^−1^ DW) (Figure 2e). In comparison to provenance BA30 (1.13 mM PRO g^−1^ DW) and BA61 (1.32 mM PRO g^−1^ DW), provenance BA60 (3.72 mM PRO g^−1^ DW) had a noticeably higher proline content among the provenances from Bosnia. Among German provenances, DE47 was superior regarding proline content (6.65 mM PRO g^−1^ DW) compared to DE48 (4.25 mM PRO g^−1^ DW) and DE49 (2.50 mM PRO g^−1^ DW).

**Figure 3:**
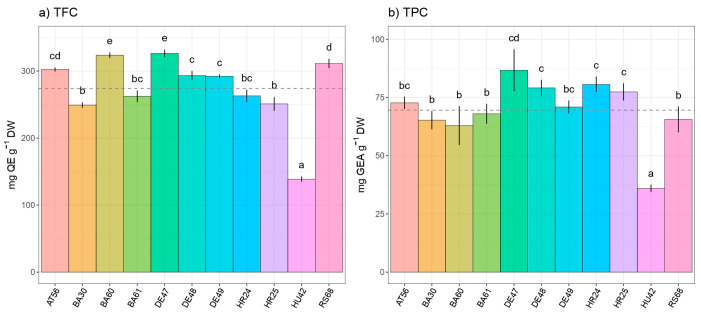
Intraspecific variability in European beech (*Fagus sylvatica* L.) leaves regarding (**a**) total flavonoid content (TFC) and (**b**) total phenolic content (TPC). Distinct lowercase letters signify significant differences observed among various species, determined through Tukey’s honestly significant difference (HSD) post hoc test (*p* ≤ 0.05). The data are presented as the mean ± standard deviation (SD).

The total flavonoids (TFC) and total phenolic content (TPC) of the inspected beech provenances differed considerably and exhibited comparable patterns of accumulation based on place of origin. As a result, the leaves of the German provenance DE47 exhibited the highest TFC and TPC contents, measuring 86.6 mg QE g^−1^ DW (Figure 3a) and 326.4 mg GAE g^−1^ DW (Figure 3b), respectively. Conversely, the Hungarian provenance HU42 demonstrated significantly lower TFC (35.9 mg QE g^−1^ DW) and TPC (138.6 mg GAE g^−1^ DW) compared to the other provenances. Provenance BA60 stood out within Bosnian provenances regarding flavonoid content, while there was no intraprovenance variability in total phenolic content among Bosnian provenances.

**Figure 4:**
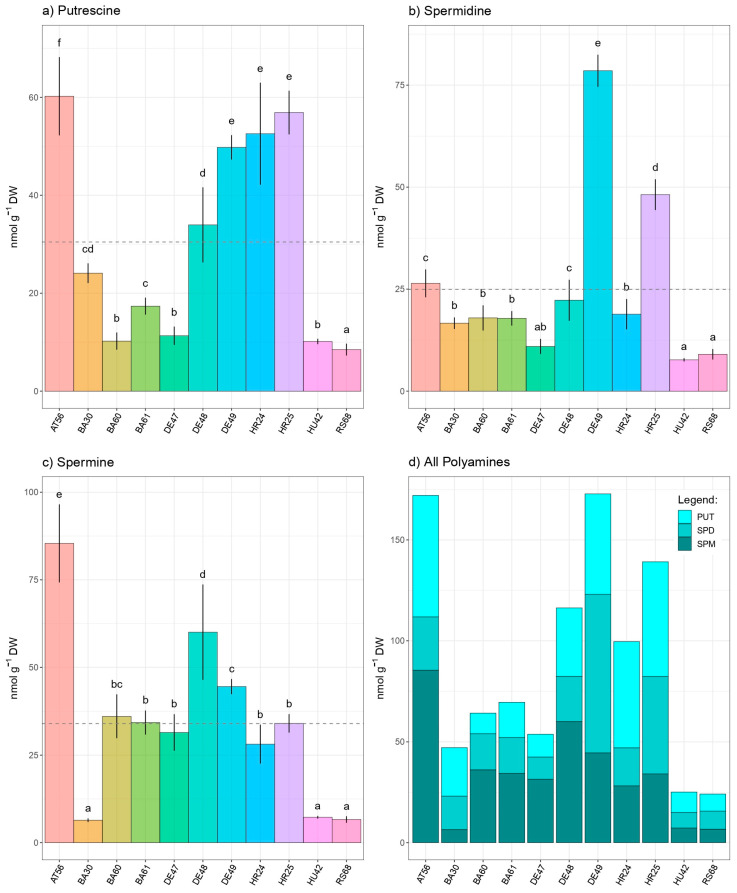
Intraspecific variability in European beech (*Fagus sylvatica* L.) regarding foliar: (**a**) putrescine (PUT), (**b**) spermidine (SPD), (**c**) spermine (SPM), and (**d**) total PAs were analyzed. Distinct lowercase letters denote significant differences observed among various species, determined through Tukey’s honestly significant difference (HSD) post hoc test (*p* ≤ 0.05). The data are presented as the mean ± standard deviation (SD).

Among all inspected parameters, individual and total polyamines (PAs) exhibited the highest inter- and intraprovenance variabilities. The highest amount of total PAs was noted in Austrian AT56 and German DE49 provenances (Figure 4d). The profiles of individual polyamines varied greatly, so in some provenances, such as HU42, HR24 and BA30, putrescine was the most dominant polyamine, while in others, such as AT56, BA60, BA61, and DE48, spermine was the most abundant polyamine. Interestingly, German provenance DE49 stood out as a provenance with prominent spermidine content. Putrescine levels in examined provenances varied from 8.48 nmol g^−1^ DW, found in Serbian provenance RS68, to Austrian provenance AT56, which had the highest putrescine level (60.2 nmol g^−1^ DW) (Figure 4a). Spermidine levels were found to be highest in German provenance DE49 (78.5 nmol g^−1^ DW), which had three times higher SPD levels than the average, and the lowest were detected in Hungarian beech HU42 (7.67 nmol g^−1^ DW) (Figure 4b). Spermine (SPM) content ranged from 6.40 nmol g^−1^ DW (BA30) to 85.4 nmol g^−1^ DW, found in Austrian provenance AT56 (Figure 4c). Furthermore, significant intraprovenance variability in all examined polyamines was recorded within Bosnian and German provenances. The total polyamine content of German provenance DE49 was three times higher than that of DE47, while Bosnian provenance B51 had levels that were 50% higher than those of B30.

**Figure 5:**
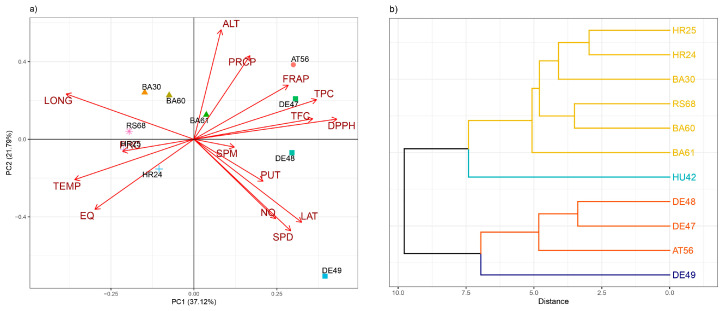
PCA analysis of all examined parameters in different beech provenances (**a**) and hierarchical clustering of 11 analyzed beech provenances (**b**). The examined parameters are abbreviated as follows: TPC (total phenolic content), TFC (total flavonoid content), FRAP (ferric reducing antioxidant power), SPD (spermidine), SPM (spermine), PUT (putrescine), RSC DPPH (radical scavenger capacity against 2,2-diphenyl-1-picrylhydrazyl radical), RSC NO (radical scavenger capacity against NO radicals), RSC ABTS (radical scavenger capacity against 2,2′-azinobis(3-ethylbenzothiozoline)-6-sulfonic acid, ABTS^•+^), ALT (altitude), LAT (latitude), LONG (longitude), PRCP (annual precipitation), and EQ (Ellenberg quotient).

According to the Principal Component Analysis (PCA) (Figure 5a), most of the inspected beech provenances were placed into the III and IV quadrants. The first two principal components described 64.64% of provenance variability. Parameters associated to the PC1 (explaining 44.66% of total variation) were antioxidant capacities such as ABTS, FRAP, and flavonoid content (TFC), while parameters SPD, PUT, and RSC NO were displayed through PC2 (explaining 19.18% of total variation). German beech provenances DE47, DE48, and DE49 separated across PC2 axes that correspond with putrescine and spermidine contents and level of NO inhibition; likewise for Bosnian provenances BA60 and BA61, with the exception of B30. Hierarchical clustering has been used to calculate and display the distance between the inspected provenances (Figure 5b).

**Figure 6:**
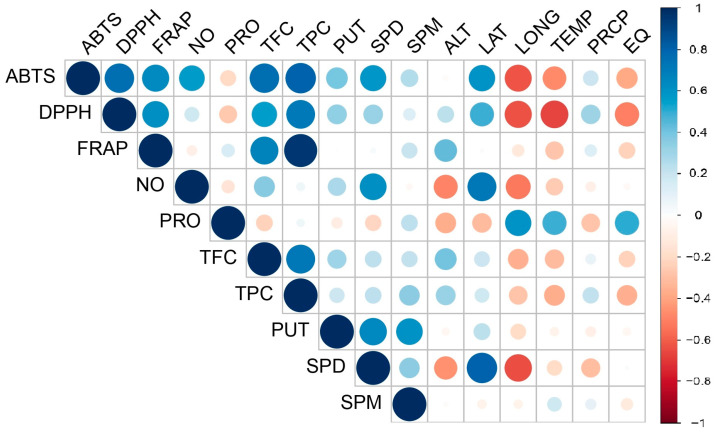
Pearson’s correlation coefficient matrix illustrates the relationships among various biochemical and bioclimatic parameters across different beech provenances. In the visualization, blue squares denote highly significant correlations between the parameters, while red squares indicate low interaction as determined by the corresponding Pearson’s coefficient. The abbreviations for the examined parameters are as follows: TPC (total phenolic content), TFC (total flavonoid content), FRAP (ferric reducing antioxidant power), SPD (spermidine), SPM (spermine), PUT (putrescine), RSC DPPH (radical scavenger capacity against 2,2-diphenyl-1-picrylhydrazyl radical), RSC NO (radical scavenger capacity against NO radicals), RSC ABTS (radical scavenger capacity against 2,2′-azinobis(3-ethylbenzothiozoline)-6-sulfonic acid, ABTS^•+^), ALT (altitude), LAT (latitude), LONG (longitude), PRCP (annual precipitation), and EQ (Ellenberg quotient).

A Pierson correlation matrix (Figure 6) outlines a strong positive correlation established between parameters describing total antioxidant power (ABTS, FRAP, and DPPH) and total phenolic and flavonoid compounds (TPC and TFC). The content of free proline was negatively correlated with TFC. Furthermore, proline content was also negatively correlated with PUT and SPD. Total antioxidant capacity estimated by DPPH and ABTS was positively correlated with all polyamines, especially spermidine. Additionally, spermidine (SPD) was highly positively correlated with neutralization of NO radicals. Interesting relationships have been found between the geographical characteristics of the beech origin (latitude, longitude, and altitude) and bioclimatic factors (precipitation, mean temperature, and Ellenberg quotient) and antioxidant and osmolyte amounts. While longitude, temperature, and EQ showed negative correlations with putrescine, ABTS, and DPPH values, precipitation, altitude, and latitude showed a mild positive correlation with putrescine and total antioxidant capacities estimated by DPPH and ABTS assays. Altitude, latitude, and precipitation had a moderately negative influence on proline levels, but temperature, longitude, and EQ showed strong positive correlations with proline. A strong negative correlation was found between altitude and longitude and spermidine levels, whereas spermidine and NO inhibition were positively correlated with latitude.

## 4. Discussion

For many decades, international provenance trials have been a hot topic in the forestry industry, particularly in relation to the European beech species, whose xeric limits have already been altered by climate change [4]. The adaptability of beech provenances with different origins and collected from various latitudes, longitudes and altitudes that were inspected in this research has been estimated through analysis of different phenological [58]. morphological [59], leaf shape, physiological and phenotypic traits. The observed variability among provenances in physiological and leaf anatomical traits was attributed to differences in the genetic architecture of the studied provenances as well as the effect of “locality” and “provenance x locality interaction” [60]. Extensive genetic diversity among beech provenances and its correlation with phylogeographical patterns has been previously confirmed through genotyping based on allozyme variation [61]. Later on, molecular markers like nuclear EST microsatellites (EST-SSRs), SNPs, and chloroplast microsatellites were also used to confirm the genetic diversity amongst beech provenances [62]. By using an untargeted metabolomic approach, three beech provenances with different latitudinal origins were distinguished for different metabolic profiles of organic and amino acids, as well as differences in levels of phenolic compounds such as caffeic and ferulic acids or kaempferol [17,19]. To the best of the authors’ knowledge, this is the first study that examines inter- and intraprovenance variability in regard to antioxidant properties and the metabolic profiles of polyamines and proline levels as important osmolytes.

Due to the wide range of physiological processes and biological functions that polyamines, as small, aliphatic, and polycationic molecules, exhibit, including flower development, embryogenesis, organogenesis, senescence, and fruit maturation and development, their levels are closely linked to the phenological phases of the species, and their endogenous levels depend on the phenological phase [63,64]. Above all, polyamines play a significant role in both biotic and abiotic stress due to their protective properties, so their biosynthesis is often triggered by various stresses [65]. The endogenous concentrations of polyamines are largely influenced by their biosynthesis, facilitated by the enzymatic actions of ornithine and arginine decarboxylase (ODC or ADC), as well as spermine and spermidine synthase (SPDS and SPMS) [66]. Additionally, their catabolism is regulated by diamine and polyamine oxidases (DAO and PAO) [66]. Furthermore, the distribution patterns of polyamines are frequently organ- and tissue-specific [67]. Furthermore, polyamine distribution also exhibits cell specificity and even cell compartment specificity since it was reported that putrescine is more prone to accumulate in the cytoplasm, while spermidine is more prone to accumulate in the cell wall [68]. Different abiotic stressors, such as drought and heat, significantly affect the expression patterns of polyamine biosynthetic genes [69,70]. Furthermore, endogenous levels of polyamines are extremely dependent on the levels of their conjugation because polyamines form numerous conjugates—mostly with phenylpropanoids to create phenolamides or hydroxycinnamic acid amides (HCAAs) due to the activity of hydroxycinnamoyltransferases [71]. Intriguingly, certain conjugated forms exhibit higher antioxidant potential than their free forms [71].

In our investigation, we found that polyamine levels varied widely throughout beech provenances, and polyamine profiles varied even more intriguingly. The detected polyamine amounts in different beech provenances were in a similar concentration range as previously reported in other woody plant species [21,22,34]. In most of the examined provenances, including HU42, HR24, and BA30, putrescine was the dominant polyamine, followed by spermidine and spermine, which is consistent with the previously established sequence in woody plant species [21,22,67]. Spermine was the most prevalent and abundant polyamine in the other beech provenances that were investigated, including AT56, BA60, BA61, and DE48. This can be explained by the so-called “polyamine cycle”, which shows that the three major polyamines are interconvertible [72]. One of the primary functions of polyamines is related to the generation of their catabolic intermediates, such as nitric oxide and hydrogen peroxide, which can both initiate the stomata closure process during heat and drought stress [32]. Furthermore, provenances with elevated polyamine levels are more resilient because polyamines are a bioenergetic smart switch for plant defense and light stress [73]. The three main polyamines showed a positive correlation, as shown by the Pierson correlation matrix, with biochemical assays for total antioxidant capacities (such as the DPPH and ABTS assays), highlighting the polyamines’ antioxidant quality that has been previously documented [22,30]. Furthermore, both total antioxidant capacities (estimated by DPPH and ABTS) and polyamines exhibited a high or mild negative correlation with EQ, which means that higher amounts of polyamines and higher antioxidant capacities are more prone to provenances that originated from lower EQ, which is known to be more favorable for beech provenances’ survival [41,42].

Significant variation was also observed in proline levels among beech provenances, with German provenance DE47 exhibiting the highest levels of this multifunctional amino acid. Proline levels found in beech provenances were in the micromolar concentration range, which is consistent with previous findings [15,21,22,23,24,34]. Proline is a particularly significant marker of plant adaptability because, in addition to its antioxidant and osmoprotective qualities, it functions as a signaling molecule and regulates a number of cellular functions, including osmotic pressure, nutrient availability, energy status, alterations in redox balance, and pathogen defenses [20]. Moreover, proline has the potential to regulate gene expression, plant development, and stress tolerance [20]. In this study, proline exhibited a negative correlation with putrescine and spermidine, which can be explained by the fact that polyamines, especially putrescine and proline, share a mutual intermediate, ornithine, so these pathways have antagonistic directions [74]. Proline levels exhibited a moderate negative correlation with altitude, latitude, and precipitation but a strong positive correlation with temperature, longitude, and EQ, indicating that provenances that originated from higher altitudes contained lower levels of proline, while those that originated from higher EQ had less abundant amounts of this amino acid.

The total phenolic content (TPC) and flavonoid content (TFC) of beech provenances showed minimal variation, but these parameters showed a strong positive correlation with assays for total antioxidant and reducing capacity (ABTS, DPPH, and FRAP). This confirmed a previously established positive correlation between these assays, which was based on the fact that they shared the same electron-transfer mechanism [75].

## 5. Conclusions

Our results showed the existence of substantial variation among European beech provenances regarding antioxidant properties and osmolyte content. A high positive correlation between individual polyamines, especially spermidine and assessed antioxidant capacities estimated by DPPH and FRAP assays, has been established, as well as among total phenolic and flavonoid content and other electron transfer (ET)-based antioxidant assays, such as FRAP, DPPH, and ABTS. Moreover, German provenance DE49 and Austrian provenance AT56 stood out regarding total polyamine content among other analyzed provenances, whereas AT56 was the most abundant in putrescine levels, while DE49 was distinguished with the highest spermidine levels. Another German provenance, DE47, had significantly higher (two-fold higher than average) levels of free proline, further suggesting that these parameters can be valuable indicators of adaptation triggered by the more stressful climatic conditions prevailing in Southern Europe. However, before declaring the aforementioned beech provenances to be “climate-ready” for migration-assisted afforestation programs in climate-smart forestry, they would be good candidates for additional genetic and transcriptome analysis of expression patterns of genes involved in polyamine and phenolic metabolism.

## Data Availability

Data are available only to Institute’s internal library database.

## Supplementary Materials

The following supporting information can be downloaded at: https://www.mdpi.com/article/10.3390/antiox13020227/s1, Table S1: Data on European beech (*Fagus sylvatica* L.) provenances in the study. Supplementary Material S2: General information and physical properties of the soil are presented for the soil depth of 30 cm.
