# Supplementary material for: Variations in Proline Content, Polyamine Profiles, and Antioxidant Capacities among Different Provenances of European Beech (Fagus sylvatica L.)
Source: Antioxidants (Basel). 2024 Feb 12;13(2):227. doi: 10.3390/antiox13020227 (PMC10886255; doi:10.3390/antiox13020227)
Supplement: Supplementary file 1 [file antioxidants-13-00227-s001.zip › antioxidants-2810624-supplementary.pdf]

Table S1. Data on European beech (*Fagus sylvatica* L.) provenances in the study.

Code	Provenance	Country	Alt	Lat	Long	T	P	EQ
HR24	Sjeverni Dilj Čaglinski	Croatia	398	45°17′	18°01′	11.9	753	29.2
HR25	Vrani Kamen	Croatia	432	45°37′	17°19′	11.1	840	25.2
BA30	Tajan	Bosnia and Herzegovina	706	44°23′	18°03′	9.6	972	20.1
HU42	Valkonya	Hungary	217	46°30′	16°45′	11.3	784	27.4
DE47	Schelklingen	Germany	675	47°59′	09°59′	9.3	952	19.6
DE48	Höllerbach	Germany	597	49°01′	13°14′	7.4	816	20.7
DE49	Hasbruch	Germany	30	53°08′	08°26′	9.6	772	22.9
AT56	Scharnstein, Mitterndorf	Austria	536	47°54′	13°58′	6.4	1473	10.4
BA60	Tešanj, Crni Vrh	Bosnia and Herzegovina	507	44°33′	17°59′	10.9	957	21.9
BA61	Bosanska Krupa	Bosnia and Herzegovina	537	44°46′	16°16′	9.8	1254	15.6
RS68	Fruška Gora	Serbia	370	45°10′	19°55′	11.0	782	27.4

Legend: Alt – altitude (m a.s.l.); Lat – latitude (N); Long – longitude (E); T – mean annual temperature (°C); P – annual sum of precipitation (mm); EQ – Ellenberg's climate quotient.

Supplementary material S2. General information and physical properties of the soil are presented for the soil depth of 30 cm.

SOIL PROPERTIES

General information*

Soil type: Cambisol
Organic carbon density: to 236 hg/m^3
Soil organic carbon stock: 52 t/ha

Physical properties*

Bulk density: 126 cg/cm^3
Clay content: 214 g/kg
Coarse fragment: 148 cm^3/dm^3
Sand content: 461 g/kg
Silt content: 325 g/kg
Volume water content at -10 kPa: 367 10^{-2} cm^3/cm^3
Volume water content at -33 kPa: 270 10^{-2} cm^3/cm^3
Volume water content at -1500 kPa: 139 10^{-2} cm^3/cm^3

Chemical properties:

Cation exchange capacity (at Ph 7)): 164 mmol(c)/kg
Nitrogen content: 178 cg/kg
Soil organic carbon content: 194 dg/kg
pH value: 5.2
